# Current Strategies in Photodynamic Therapy (PDT) and Photodynamic Diagnostics (PDD) and the Future Potential of Nanotechnology in Cancer Treatment

**DOI:** 10.3390/pharmaceutics15061712

**Published:** 2023-06-12

**Authors:** Marta Olszowy, Martyna Nowak-Perlak, Marta Woźniak

**Affiliations:** Department of Clinical and Experimental Pathology, Division of General and Experimental Pathology, Wroclaw Medical University, 50-368 Wroclaw, Poland; marta.olszowy@student.umw.edu.pl (M.O.); martyna.nowak-perlak@student.umw.edu.pl (M.N.-P.)

**Keywords:** photodynamic therapy, nanotechnology, cancer, combined cancer therapy, quantum dots, photodynamic diagnostic

## Abstract

Photodynamic diagnostics (PDD) and photodynamic therapy (PDT) are well-established medical technologies used for the diagnosis and treatment of malignant neoplasms. They rely on the use of photosensitizers, light and oxygen to visualize or eliminate cancer cells. This review demonstrates the recent advancements in these modalities with the use of nanotechnology, including quantum dots as innovative photosensitizers or energy donors, liposomes and micelles. Additionally, this literature review explores the combination of PDT with radiotherapy, chemotherapy, immunotherapy, and surgery for treating various neoplasms. The article also focuses on the latest achievements in PDD and PDT enhancements, which seem to be very promising in the field of oncology.

## 1. Introduction

The photodynamic reaction is a well-established phenomenon that has served as a diagnostic tool and a therapeutic approach for diverse neoplasms. The general idea of combining specific plants extracts with phototoxic properties, along with their constituent chemicals, has been around for centuries. The first scientific description of photodynamic therapy (PDT) was introduced by Hermann von Tappeine in 1904, who conducted experiments with the topical application of photosensitive dye to treat various skin diseases, followed by light exposure. The photodynamic reaction is based on an interaction between a photosensitizer, oxygen, and light with an appropriate wavelength [1,2]. The photosensitizer, upon absorbing light, initiates the photochemical reactions that can lead to cell visualization or destruction by generating reactive oxygen species (ROS) that have accumulated from the photosensitizer. Both of the above-mentioned mechanisms are thoroughly described in subsequent sections of this review.

Photodynamic diagnostics (PDD) can be divided into the following two sections: purely diagnostic, when the application of a photosensitizer enables the visualization of cancerous tissue, thus allowing us to provide an accurate diagnosis, and therapeutic, which involves visualizing cancerous tissue, leading to its subsequent elimination, usually by surgical removal or subsequent PDT [3,4].

Despite the wide variety of studies proving the effectiveness of PDT, there is still room for improvement. One of the promising directions is an approach combining photosensitizers with nanotechnology, in order to increase their affinity towards tumor cells and excel the delivery process. It is well-established that nanodrug-based therapies solve some of the most intractable problems in cancer treatment, such as tumor metastasis and drug resistance. The use of photodynamic therapy and nano-PS allows for precise drug release, efficient response to light stimuli and the ability to overcome hypoxia-induced resistance. Moreover, the development of nano-PS enhances the generation of ROS and the overall efficacy of PDT. Although PDT is often used alone, as a single therapy, displaying numerous benefits for neoplasm treatment, recent studies have focused on combining conventional and innovative methods to target malignant neoplasms. This strategy combines a unique mode of action against cells, with the potential for additive or even synergistic effects. Research has revealed enhanced effectiveness by either pre-attenuating tumor cells to make them more receptive to subsequent PDT or by minimalizing the survival mechanisms of cells resistant to PDT [5].

In the last ten years, a significant number of reviews describing similar topic to ours [2,5,6,7,8,9] have been published. This review differs from previous publications in several ways.

Most of the reviews heavily focus on the principal of mechanism of action of PDT. Only a few mention the usage of nanoparticles in combination with photosensitizers [6], and the topic of PDD is narrowly described [6,7]. Many publications focus solely on the treatment and diagnosis of one type of tumor [3,10,11,12].

In our review, we focus on the new and rising approaches in PDT and PDD, focusing on combining PDT with different treatment methods, as well as combining photosensitizers with nanoparticles. We summarize the diagnostic approach of PDD, describing the most widely studied photosensitizers for PDD.

In this review, our objective was to investigate the latest achievements of the photodynamic reaction efficacy in neoplasm diagnostics with a broad spectrum of photosensitizers and novel delivery methods based on quantum dots, liposomes and nano-drugs. We focused on the in vitro and in vivo studies, as well as clinical trials. Among the most popular researched photosensitizers presented for PDD were 5-ALA, indocyanine green, sodium fluorescein, porfimer sodium, talaporfin sodium, O-chlorin and G-chlorin. Moreover, the goal of the article was to provide updated insights on combined photodynamic therapy with different methods used to treat malignances. Despite many encouraging clinical results in patients’ treatment, PDT still remains clinically underutilized as a standard method in oncology. This highlights the need for further studies to evaluate the efficacy of PDT in combination with immunotherapy, radiotherapy, chemotherapy, and surgery.

## 2. Photodynamic Therapy

### 2.1. The Principle of PDT

Photosensitizers are a group of substances that share similar properties. When the light interacts with a photosensitizer’s molecule, the molecule (singlet state) absorbs energy from the wavelength and gets excited into an excited singlet state. This arrangement is imbalanced, so a way of giving the energy away is found. Part of the energy is emitted in the form of the fluorescence and the photosensitizer is directed to the excited triplet state. The remaining energy is further given away in two ways.

In the type one reaction, energy is transferred into surrounding molecules in the form of electrons or hydrogen, which leads to the creation of free radicals and anion radicals of the photosensitizer and the substrate. The electron reacts with oxygen molecules, which creates superoxide anion radicals (O2^•−^) and further generation of ROS (reactive oxygen species). This cascade leads to the oxidative stress that is responsible for the destruction of cancer cells.

The type two reaction is slightly different to type one. Energy from the excited triplet state photosensitizer is transferred directly to the oxygen molecule, thus creating singlet oxygen, which has very strong oxidizing properties. The photosensitizer then returns to its original state.

**Figure 1:**
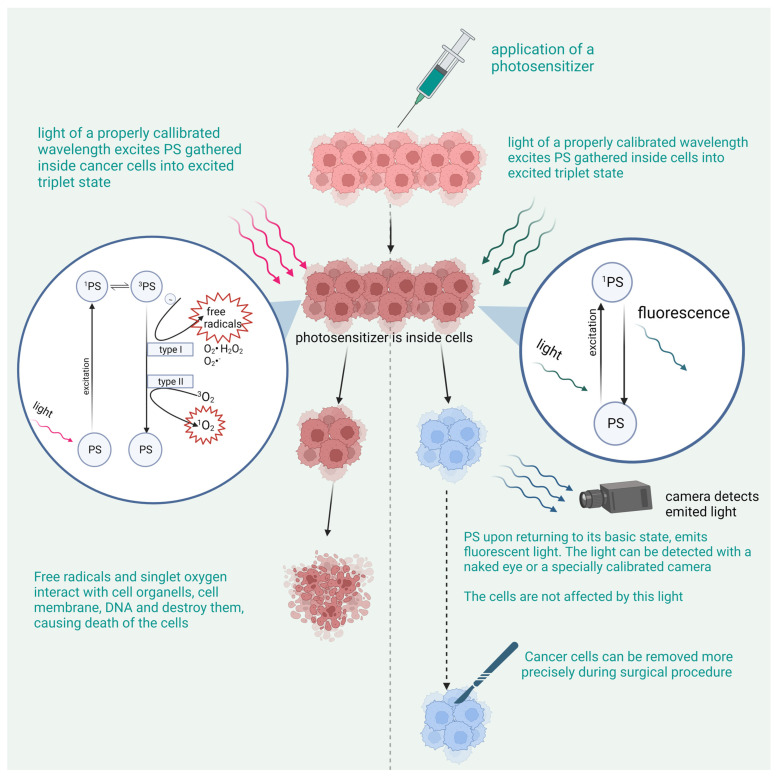
The mechanism of action of PDT and PDD.

ROS and singlet oxygen cause oxidation of the cell structures, leading to their destruction. PDT also stimulates the immune system to act against cancer. The antitumor effect is also achieved by the occlusion of blood vessels around the tumor (Figure 1).

One particular ability of the photosensitizers that makes them useful in terms of therapy is their tendency to accumulate in cancerous cells. It is suspected that this is due to their preference to combine with LDL (low-density lipoproteins), which are absorbed in significant amounts by cancerous cells (they need cholesterol that is transported by LDL so they absorb LDL in a greater amount than normal cells). Moreover, PSs accumulate for a longer time due to better vascularization and blood flow in a cancer microenvironment, in comparison to normal tissues. This natural tendency of photosensitizers allows for their use in PDT and PDD. However, this mechanism is not perfect, and some part of the photosensitizer also accumulates in normal cells. Therefore, scientists are developing ways to improve the intracellular accumulation process [5,8,9].

#### The Mechanism of the Cancer-Killing Process

The most direct mechanism of the PDT-induced anticancer effect is lethal oxidation in the cancerous cells. ROS and singlet oxygen particles react with cell organelles and DNA, leading to their destruction. Another way that PDT works is by stimulating an immune response. Destruction of organelles and the membrane of cancer cells leads to the activation of phospholipases and cyclooxygenases, further causing the release of inflammatory mediators that attract leukocytes. Leukocytes then start to act against already destroyed and remaining cancerous cells, and the antivascular effect of PDT is achieved within tumor tissue. Photodestruction of the vessel walls also attracts macrophages and neutrophils [9].

However, Gomer et al. describe the opposite mechanism of interaction between PDT and the immune system that occurs in the tumor microenvironment. The authors suggest that the death of tumor cells and the secretion of mediators of the immune reaction by them stimulates leukocytes that have been previously recruited by the tumor to release COX and prostaglandins, as well as other mediators that stimulate angiogenesis. Hypoxia, which occurs in the tumor area after PDT, has a similar effect—it also leads to the stimulation of angiogenesis. The authors suggest combining PDT with angiogenesis inhibitors [13].

### 2.2. Combining Photosensitizers with Nanotechnology Help Improve PDT

**Figure 2:**
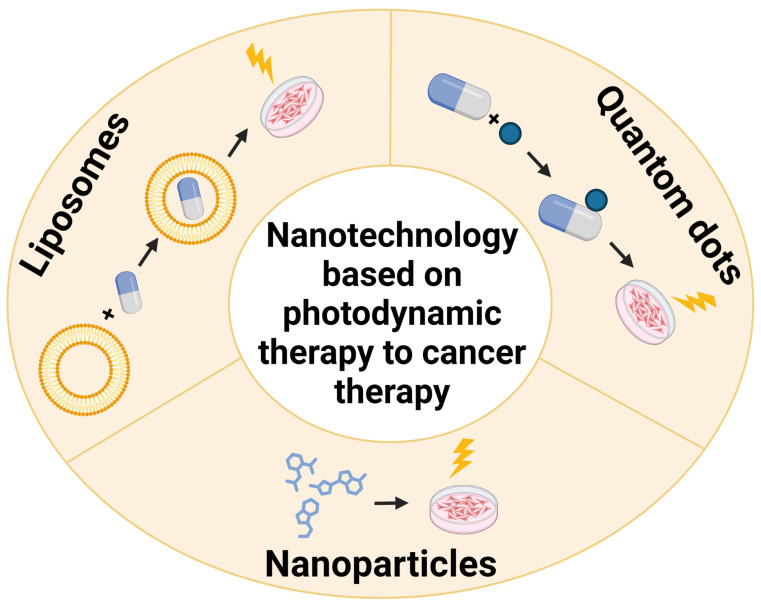
Nanotechnology based on photodynamic therapy fields of interests.

Within the realm of nanoparticles, this review focuses on quantum dots, nanodrugs and liposomes (Figure 2).

#### 2.2.1. Quantum Dots and PDT

Quantum dots (QDs) are nanoparticles, which are promising in biology, medicine, and industrial use. The wide-ranging biological applications of QDs are due to their unique particle size, excellent photo-physical properties, luminescent features, and biocompatibility. Interestingly, the size of QDs helps to impart unique optical (high brightness, high quantum yield, high absorption factor, intermittent fluorescence signals) and electrical features [14]. QDs can be created using a variety of methods, including colloidal quantum dots for use in biological systems, as well as self-assembling nanoislands on surfaces that can be used as single-photon transmitters in electronics for information storage. Some studies confirmed that QDs exhibit promising outcomes in PDT due to their capability to act as effective photosensitizers or energy donors to other photosensitizers. Upon irradiation, QDs generate reactive oxygen species (ROS), which play a crucial role in inducing cell death during cancer treatment [15,16,17].

Zhang et al. created PC@BPQDs, or black phosphorus quantum dots with phycocyanin (PC) functionalization. A strong photosensitizer known as black phosphorus (BP) has been created for phototherapy due to its favorable optical features, photothermal effects, and photodynamic effects. The PC layer efficiently reduces plasma protein adsorption onto BPQDs while also functionally increasing the effectiveness of photothermal therapy through improved ROS release, which increases in vitro apoptosis. The PC@BPQDs are more stable in vitro and in vivo than PC and BPQD alone, retaining their photothermal effect and photodynamic activity to the fullest extent possible. The experiments established that the PC corona of PC@BPQDs is a protective barrier that prevents plasma proteins from adhering to the BPQD surface and supports the stability of the nanoparticles. They discovered that PC@BPQD nanoparticles can specifically accumulate in tumor tissues, which facilitates their biomedical functions. They also discovered that PC@BPQD nanoparticles can efficiently enter tumor cells through endocytosis and escape from the endosomal/lysosomal compartments into the cytosol. Additionally, PC@BPQDs eliminate tumors in vivo with good efficacy and minimal toxicity. Therefore, PC@BPQDs have a bright future in terms of therapeutic applications [18]. Hashemkhani et al. created cetuximab-Ag2S quantum dots (QDs) to treat colorectal cancer (CRC). In this experiment, 5-ALA was loaded with QDs in three different ways to trigger its release via different stimulants. Due to improved particle absorption, rapid ALA release, and efficient 5-ALA-to-PpIX conversion, the most effective phototoxicity was observed in SW480 cells when using AS-2MPA-ALA-electrostatic-Cet. Targeted administration substantially decreased the effective 5-ALA concentration, which was further decreased with 5FU delivery. Although the incubation period for the release of 5-ALA in therapeutic amounts was prolonged, covalent linkage delivery of 5-ALA was still efficacious for PDT. High amounts of reactive oxygen species (ROS) and apoptotic/necrotic cell death were linked to phototoxicity. The scientists have not observed a loss of stability of the created substance, which may result in the strength of the bonds. The approach of these scientists provides a new and highly effective theranostic method for the treatment of colorectal cancer [19].

#### 2.2.2. Nanoparticles and PDT

Nanoparticles have emerged as a possible solution to the obstacles of anti-cancer drug delivery. They are tiny molecules with distinct physiochemical characteristics that range in size from 1 to 100 nm. Nanoparticles are significantly smaller than cells, they can interact with the cell membrane and be taken within the cell, making them highly important in biomedicine [20]. Their easy and reproducible preparation technique helps remove many hurdles, including quality control, scale-up production and clinical trials. Nanoparticles can act as both carriers and cargoes for drugs [21,22,23].

Hosseinzadeh et al. employed methylene blue–curcumin ion pair nanoparticles and single dyes as photosensitizers for the evaluation of photodynamic therapy treatment on the human breast cancer cell line (MDA-MB-231 cell line). The outcomes reveal that the ion pairing of methylene blue and curcumin improves the photodynamic activity of both dyes and the cytotoxicity effect of ion pair nanoparticles on the MDA-231 cell line. Blue and red LED light sources were used for the photoactivation of the photosensitizers. Additionally, red light can activate both dyes more effectively than blue light when generating singlet oxygen [24]. Guo et al. developed a pH/glutathione (GSH)-responsive nano micelle (NLG919/PGA–Cys–PPA@Gd) with a high drug-loading ratio and controlled drug release performance. The scientists prepared this nano-drug for T1-weighted MRI-guided PDT and immune synergistic therapy. The outcomes demonstrated that under typical physiological conditions, micelles had a significant drug loading rate. The results from the experiments also indicate that created nanomicelles have long-term stability under normal physiological conditions. Furthermore, created micelles could avoid the undesired PPA leakage in blood circulation due to the conjugation between PPA and polymers. Due to their high relaxivity, the produced micelles may also improve the contrast of lesions in T1-weighted MRI scans. The in vivo results showed that the developed nano drug displayed substantial accumulation in tumor locations and successfully damaged cancer cells. Moreover, the micelles had good biocompatibility and biosafety during in vitro and in vivo assays. The scientists suggested nano drug micelles as an effective approach for MRI-guided PDT and immune synergistic therapy [25].

#### 2.2.3. Liposomes and PDT

To improve the bioavailability of photosensitizers (PSs) in photodynamic therapy, PSs can be encapsulated in liposomes. These excellent multi-functional nano-carriers for drug delivery have been successfully used for the past 30 years. Liposomes have demonstrated the ability to enhance drug permeability into biological membranes, better tissue penetration than normal PS, and have higher tumor specificity. Additionally, liposomes can close hydrophobic and lipophilic agents. Furthermore, the flexibility of surface modification and liposome components provide benefits in targeted drug delivery, controllable drug release, image-guided treatment, and combined therapy. Polyethylene glycol (PEG) is the most often employed surface coating component for liposomes currently [26,27,28].

Plenagl et al. conducted research on human ovarian adenocarcinoma cells (SK-OV-3). The scientists encapsulated hypericin by the thin film hydration method and a hypericin-hydroxypropyl-β-cyclodextrin inclusion complex (Hyp-HPβCD) was incorporated by the dehydration–rehydration vesicle method in either conventional or ultra-stable tetraether lipid (TEL) liposomes. The stability of liposomes was confirmed by incubation in cell culture media. Filtration with a 0.22 m nylon filter purified the Hyp-HPCD complex solution. Confocal microscopy was applied to show the absorption of liposomal hypericin into the SK-OV-3 cell line. It was demonstrated that the lipid composition and intraliposomal hypericin localization influenced the anti-vascular influence in the chorioallantoic membrane (CAM). Although hypericin TEL liposomes significantly destroyed the microvasculature, drug-in-cyclodextrin TEL liposomes had no impact. Interestingly, in a safe dose range, both formulations caused significant photocytotoxicity in SK-OV-3 cells. They suggested additional research into liposomal release properties and determining the preferred accumulation location in tumor tissue in order to develop a liposomal combination therapy for safe and targeted anticancer therapy [29]. Woźniak et al. conducted experiments that were focused on the investigation of curcumin encapsulated in liposomes and its increased photoactive properties in photodynamic therapy. In their study, curcumin was encapsulated in liposomes made of hydrogenated soy phosphatidylcholine (HSPC), which demonstrated high stability due to a highly stiff bilayer and, as a result, limited curcumin diffusion characteristics. They conducted research on melanoma (MugMel2), squamous cell carcinoma (SCC-25), and normal human keratinocytes (HaCaT) cell lines. The cancer cells revealed increased phototoxicity after the therapy in comparison to normal cells. Furthermore, liposome curcumin-based photodynamic therapy improved the ratio of apoptotic to necrotic cells. The research additionally showed that liposome curcumin significantly reduced malignant cell motility after PDT therapy. The findings indicate that liposomal encapsulation of a poorly soluble natural chemical may increase the photosensitizing effects of curcumin-mediated PDT therapy in skin malignancies while reducing toxicity in normal keratinocytes [30]. Feuser et al. evaluated the synergistic effect of diethyldithiocarbamate (DETC) and zinc phthalocyanine (ZnPc) co-encapsulated in liposomes in photodynamic therapy (PDT). The liposomes were stable for 40 days, according to stability studies. The liposomes provided greater protection to the drugs when exposed to non-tumor cells, which were mouse embryo fibroblast (NIH3T3) cells. It is important to note that both the free drugs and co-encapsulated drugs in liposomes promoted more pronounced phototoxic effects on human breast cancer cells (MDA-MB231) compared to the treatment with ZnPc alone. The researchers showed that the encapsulation of ZnPc and DETC significantly inhibited the antioxidant enzyme activity of SOD and GSH in MDA-MB 231 cells. They expected that DETC decreased SOD and GSH activity, leaving more sensitive cells to be treated with irradiation. This decrease in the antioxidant activity was induced by an increase in ROS production generated by PDT and consequently resulted in greater cell death. Feuser et al. reported that these findings present a good option for breast cancer therapy with PDT, as well as a foundation for future research on varied light dosages and other biological factors [31]. Cong et al. constructed drug delivery liposomes (MnO_2_-PTX/Ce6@lips) loaded with catalase-like nanozymes of manganese dioxide nanoparticles (MnO_2_ NPs), paclitaxel (PTX) and chlorin e6 (Ce6) to consume the tumor’s native H_2_O_2_ and produce O_2_. Based on the MnO_2_ NPs’ catalysis, MnO_2_-PTX/Ce6@lips produced a significant amount of oxygen in order to burst the liposomes and release the loaded drug (paclitaxel). The oxygen that was then released reduced the tumor cells’ chemoresistance and provided the building blocks for photodynamic therapy. Then, in an acidic tumor environment, MnO_2_ nanoparticles were broken down into Mn^2+^ to be used as contrast agents for magnetic resonance imaging. The results of the energy-dispersive spectroscopy (EDS) assay and EDS elemental mapping images proved that the purity of MnO_2_, which was used to create liposomes, was very high. The scientists confirmed the anti-cancer effect of the tested compound in in vitro and in vivo assays. They conducted an MTT assay, took live/dead cell images and measured apoptosis by flow cytometry. The addition of MnO_2_ NPs improved the hypoxia environment, relieved the drug resistance of tumor cells, and provided oxygen for PDT. The chemotherapy and PDT combination has good antitumor benefits after reducing tumor hypoxia. The in vivo results showed that the growth of the tumors was well inhibited in the MnO2-PTX/Ce6@lips and irradiation. Additionally, the scientists proved that the created substance has high stability and enhances the sample’s extended circulation period in the body. Their results demonstrated MnO2-PTX/Ce6@lips’ significant potential for influencing the microenvironment and improving chemotherapy, PDT, and MRI tracking in the management of tumors [32].

### 2.3. Combination of PDT with Different Treatment Methods

PDT is often used alone, as a single therapy and shows many benefits when used to treat neoplasms. Due to the high adherence of the photosensitizers to the tumor tissue, PDT works locally. This allows for the elimination of systemic complications—a serious problem in other treatment options, for example chemotherapy and radiotherapy. Those complications can be additionally limited by carefully irradiating only the tumor. Compared to surgery, PDT can be effective against metastases. It is not an invasive procedure and does not leave scars when used topically. Usually, one-time treatment is effective, although it can be repeated when needed without any drawbacks, unlike radiotherapy. Administration of the drug is relatively easy and not unpleasant; it can be delivered per os, intravenously or topically, depending on the location and the type of the tumor. The equipment needed to perform PDT is not extremely expensive and relatively easy to use. Photosensitizers used for photodynamic therapy should not have a toxic impact on the organism. Although the advantages are strong, there is always room for improvement. Combining photosensitizers with various techniques, both already used and under study, has been researched by many scientists. In this paragraph, PDT was coupled with immunotherapy, radiotherapy, chemotherapy, and surgery.

#### 2.3.1. Immunotherapy

There are a handful of ideas of combining PDT with immunotherapy. PDT on its own stimulates an immunologic response against tumors.

##### The Use of Antibodies to Accelerate the Affinity of the Photosensitizer to the Cancerous Cells

PIT, or photodynamic immunotherapy, is based on the idea of combining antibodies targeted against specific proteins within cancer cells with a photosensitizer. That way, the affinity of the photosensitizer is increased, thus leading to more efficient PDT. Many proteins that are characterized by a higher concentration in the tumor tissue can be targeted, such as EGFR (Mab), EpCAM, and FAP [33,34]. Nanoparticles are often used in PIT, allowing us to create complexes of even higher affinity to the cancerous cells [34].

Monoclonal antibodies (Mab) can be used to enhance the photosensitizer’s affinity to the cancerous cells. Mab can be directed against EGFR, which are present in higher concentrations than in normal cells. When Mab is combined with the photosensitizers’ molecule, it becomes more specific and leads to a higher concentration of the Mab-photosensitizer in the cancerous cell. That way, more efficient PDT can be achieved [35].

##### Combining Immunological Adjuvant and PDT

Another approach is a new technique called LIT (laser immunotherapy). It is based on adding an immunological adjuvant to PDT, which allows for an improved immunological response stimulated by PDT. An example of this technique is the nanoliposome-encapsulating photosensitizer Chlorin e6 and Galectin-3 (Gal-3) inhibitor, which allows the NK response to be enhanced after PDT [34]. A similar effect can be achieved upon administrating the immunoadjuvant before the photosensitizer. Castano et al. tested whether the administration of low-dose cyclophosphamide prior to PDT with benzoporphyrin derivative monoacid ring A verteporfin could improve its anticancer effect. Low-dose cyclophosphamide has a well-documented immunoadjuvant antitumor effect in form of the depletion of T-reg cells and attenuation of their suppressive function, as well as the enhancement of effective T-cell function and a change in the cytokine secretion profile of the helper T-cells to a more anticancer type [36]. Castano et al. compared the antitumor effect against J774 cells (reticulum cell sarcoma) of BPD-PDT alone, BPD-PDT preceded by low-dose cyclophosphamide, high-dose cyclophosphamide alone and followed by BPD-PDT. The results show that only low-dose cyclophosphamide combined with BPD-PDT cured the cells and provided resistance against the cancer cells [37]. Similar outcomes were achieved by Reginato et al. upon combining low-dose cyclophosphamide with PD-PDT against CT26 cancer (mouse colon carcinoma) [38].

##### Combining PDT with a Check-Point Blockade to Stop the Tumor from Recruiting Lymphocytes for Its Microenvironment

Check-point blockade is a new promising cancer immunotherapy, based on the blockade of the tumor-associated enzymes that promote lymphocytes-T dysfunction. It has not yet been proven to be effective enough. Thus, attempts to combine check-point blockade with PDT have been made and shown improved treatment results [34]. A significant amount of research has been developed where combining check-point inhibitors, such as the IDO inhibitor, anti-CTLA4, and PD-L1 inhibitor, with photosensitizers was tested and showed promising results. Various particles were used to combine the beforementioned molecules, providing better affinity [34,39].

##### The Use of PDT to Create Anticancer Vaccines

PDT is also used to produce anticancer vaccines, by stimulating the death of cancer cells and activating dendritic cells. The cancer cells are killed using PDT and their remains are incubated with immature dendritic cells (DC). That way, prepared DC-based vaccines are injected into animals with tumors. This vaccine enhances the immune response against the tumor. Further research is needed to further develop this technique [34,40].

#### 2.3.2. Radiotherapy

Radiotherapy, although widely used and shows very good results in the treatment of many cancers, presents the following limitations: side effects such as skin burnings and resistance of hypoxic cancer cells. A few studies have been conducted, and the results showed that PDT can increase the tumor sensitivity to radiation and allow for a reduction in the dosage. Ionizing radiation, compared to visible light and NIR light, is characterized by deeper tissue penetration.

##### The Use of X-rays to Excite the Photosensitizer

One of the attempts to combine PDT and RT is X-PDT, which is a type of PDT where the energy exciting PDT comes from radiation. Using X-rays helps with the low penetration depth of visible light. A combination of a scintillator and photosensitizer is used. Scintillators exhibit X-ray-excited optical luminescence (XEOL). The scintillator interacts with the radiation photons and emits visible photons, which are able to further activate the photosensitizer. The choice of the scintillator and the photosensitizer is important as- the wavelength of the visible photons emitted by the scintillator has to be able to excite the photosensitizer. They need to be close to one another; thus, the molecules are often connected. The scintillator is usually a nanoparticle. Examples of these molecules are as follows: LaF3:Tb-MTCP; Tb2O3@SiO2-porphyrin; GdEuC12 and hypericin (Hyp); Gd2O2S:Tb and photofrin II. The amount of radiation that reaches the cancer tissue and accumulates at the scintillator–photosensitizer complex is usually more than enough to stimulate the scintillator. The excess radiation interacts with the cancer tissue, working the traditional way and leading to its death. That way, the tumor is destroyed simultaneously by the radiation and PDT. There are a few photosensitizers that do not need the scintillator to transfer the radiation’s energy to them and can be excited directly by the ionizing radiation, for example arcidine orange [41].

X-PDT, compared to RT only, is proven to be more effective. In comparison, in research using MC540-SAO:Eu@mSiO2 as the photosensitizer (combined with nanoparticles) and ionizing radiation, several factors were compared. X-PDT, when compared to RT only, showed lower cell viability and clonogenicity as well as higher necrosis and apoptosis [42]. Close investigation revealed that X-PDT works both as PDT and RT, causing different damage to the cancer, cells thus killing them more effectively [41,42].

##### The Combination of PDT and Radiotherapy

Another approach is to use PDT and RT in the same patient, but not combine them into one method. This strategy can be used only in the chosen types of cancer, where the source of light can be placed close to the cancerous tissue, such as gastric cancer, oesophageal cancer, rectal cancer, bladder cancer, and cervical cancer. Yi-shan Wang et al. researched the before-mentioned cancers in end-stage patients and co-therapy with PDT and RT, and the results showed the alleviation of symptoms and enhanced response to the radiation [33].

#### 2.3.3. Chemotherapy

Chemotherapy is a widely used anti-tumor treatment; however, it is characterized by high toxicity. Combining chemotherapeutic drugs with PDT can benefit the patients in the following two ways: improving the efficiency of the treatment and lowering side effects.

##### Combining Chemotherapy and PDT Enhances the Anticancer Properties of Chemotherapy

Combining chemotherapy with PDT is promising due to the high toxicity of chemotherapy. The precise mechanism in which this works is unknown. Combining those two techniques allows us to minimize the side effects of chemotherapy without losing its effectiveness against cancerous cells. A few combinations were tested, and the results indicate the effectiveness of a lower chemotherapy dose, the overcoming of multidrug resistance, and the therapeutic effect in late-stage patients [9]. For example, You-Shuang Chen et al. combined tNBS-COOH and cisplatin in vitro lung cancer (SCLC) treatment, demonstrating that it leads to decreased cell viability and migration when compared to PDT alone [43].

##### Combining Chemotherapy with PDT Enhances the Anticancer Effect of PDT

On the other hand, chemotherapeutic drugs can be used as PDT enhancers. In this approach, the application of chemotherapeutics prior to PDT leads to the higher sensitivity of cancer cells and higher efficiency of PDT. The most widely researched chemotherapeutic is methotrexate.

Anand et al. compared the anticancer effect of 5-ALA-PDT alone and preceded by low-dose methotrexate against several cancer types, and the results showed the increased cellular intake of 5-ALA and higher efficiency of PDT when combined with methotrexate [44]. Similar observations were made by Sinha et al. during research on 5-ALA-PDT preceded by methotrexate against LNCaP human prostate carcinoma; the cellular intake of the 5-ALA derivative, PpIX, increased three-fold and a higher efficiency of PDT was observed [45]. Salva et al. researched the enhancement of 5-ALA-PDT with prior administration of methotrexate against CTCL, cutaneous T-cell lymphoma, and described the mechanism behind the enhancement process. The study suggests that the upregulation of FAS, FASL, TRAIL and TNFα caused by methotrexate leads to the greater induction of extrinsic pathway apoptosis by 5-ALA-PDT [46,47].

##### The Use of Drugs That Have Both PS and Cytostatic Properties

Some chemotherapeutic drugs such as mitoxantrone (MX) are also photosensitizers. The use of both properties of mitoxantrone, i.e., the chemotoxicity towards the cancerous cells and the excitation by the proper wavelength of light that leads to the creation of ROS, can improve the tumor response to the treatment [34]. Zeyong Li et al. proved that mitoxantrone-filled micelles, when irradiated with a suitable wavelength of light, caused the increase in ROS. This led to the increased uptake of mitoxantrone by cancerous cells, thus overcoming MDR (multi-drug resistance) [48].

#### 2.3.4. Surgery

The concept of combining photosensitizers with the surgical removal of the tumor allows us to overcome many drawbacks. This is especially useful in those types of tumors where resection with a wide margin is not possible, for example brain tumors. The surgical removal of the tumor is accompanied by the technique called PDD or photodynamic diagnostics. PDD, although often based on the same photosensitizers, uses a different mechanism than PDT. The photosensitizer is excited with a different wavelength and, upon returning to its basic state, emits fluorescence light. Special equipment is used to detect the emitted light. The exact mechanism is described in the section on PDD. When combined with the surgical removal of the cancerous tissue, PDD allows for high precision when cutting out the tumor. The photosensitizer is usually administered to the patient before the procedure, which allows for the photosensitizer’s molecules to enter cancerous cells. Then, the surgical procedure is performed and the tumor is dissected. Then, before the removal of the tumor, the tumor and surrounding area are illuminated with a suitable wavelength and observed with a suitable camera. All cancerous tissue emits light and is easily distinguished from healthy tissue, thus allowing for higher precision during its removal.

The combinational technique of photodynamic therapy (PDT) and fluorescence-guided surgery has been extensively investigated in various types of brain cancer, especially high-grade glioma (HGG), using 5-ALA, which has been approved for clinical use [49,50]. Fluorescence-guided surgery of the pituitary gland tumors has been studied with the use of various photosensitizers, such as 5-ALA, indocyanine green or fluoresceine sodium. Despite the results varying between types of pituitary cancers, promising and further research is required in order to determine the applicability of this method in clinical settings [51,52]. The examinations of fluorescence-guided resection of the different types of brain cancer, including metastases, have yielded varying results; thus, further research is required [53]. Notably, in the case of bladder cancer, a PDD-guided surgery technique has been registered for clinical use [54,55].

Many substances have gained researchers’ attention and their usefulness in PDD-assisted surgery are being evaluated [56,57,58].

### 2.4. Photodynamic Diagnostics

A different direction of using photosensitizers is diagnostics. Many photosensitizers have predispositions for use in diagnostics and have been of interest to researchers and clinicians for many years, and research on individual substances is at various stages of advancement.

The idea to use photosensitizers in diagnostics comes from their properties. The following two main characteristics of these compounds make them useful in terms of diagnosis: their increased affinity to cancerous cells (primarily caused by the increased uptake by cancerous cells and the tendency to accumulate in the areas with increased blood perfusion), as well as the properties that make the compound visible.

## 3. The Mechanism of PDD

The visualization process uses a mechanism of fluorescence. Visible light at a specific wavelength, upon reaching the photosensitizer, gives its energy to it and activates it, causing it to become excited and turning it into a single excited state photosensitizer, which leads the photosensitizer to give away this energy by emitting a different wavelength of visible light and returning to its basic state. No other mechanisms to give away energy are triggered, and, as opposed to PDT, tissues that are nearby to the fluorescent photosensitizer are not affected at all, and no damage is carried out to the cells. This mechanism, when combined with proper tools that allow the emission and detection of appropriate wavelengths, leads to the precise visualization of malignant cells without causing any damage. This makes PDD a promising technology with a wide range of applications.

Visualization requires specialized equipment, consisting of a light source that emits light at the proper wavelength to excite the photosensitizer, as well as a detector that is able to detect light beamed by an excited dye. Practically, systems have been developed that contain a camera, a light source, an excitation module, an optic device for light perception, as well as a computer system to analyze the acquired images, and a mechanical structure to support the whole system. Many machines have been developed and calibrated to meet the requirements of a photosensitizer of which the most widely used are videoendoscopes and optical microscopes [3,59,60].

Three main ways of drug administration are as follows: per os, intravenous and topical. A photosensitizer is expected to have a higher affinity to cancerous cells, thus making it possible to visualize them with proper equipment.

The most widely researched photosensitizers are 5-ALA, indocyanine green, sodium fluorescein, porfimer sodium, talaporfin sodium and O-chlorin (Table 1).

The majority of the presented compounds have been known and present in the medical field for many years. Looking for new usages of known substances comes with many advantages, such as eliminating expensive trials of its impact on the human body, the cost of developing suitable equipment and the registration process.

### 3.1. 5-Aminolevulinic Acid (5-ALA)

**Figure 3:**
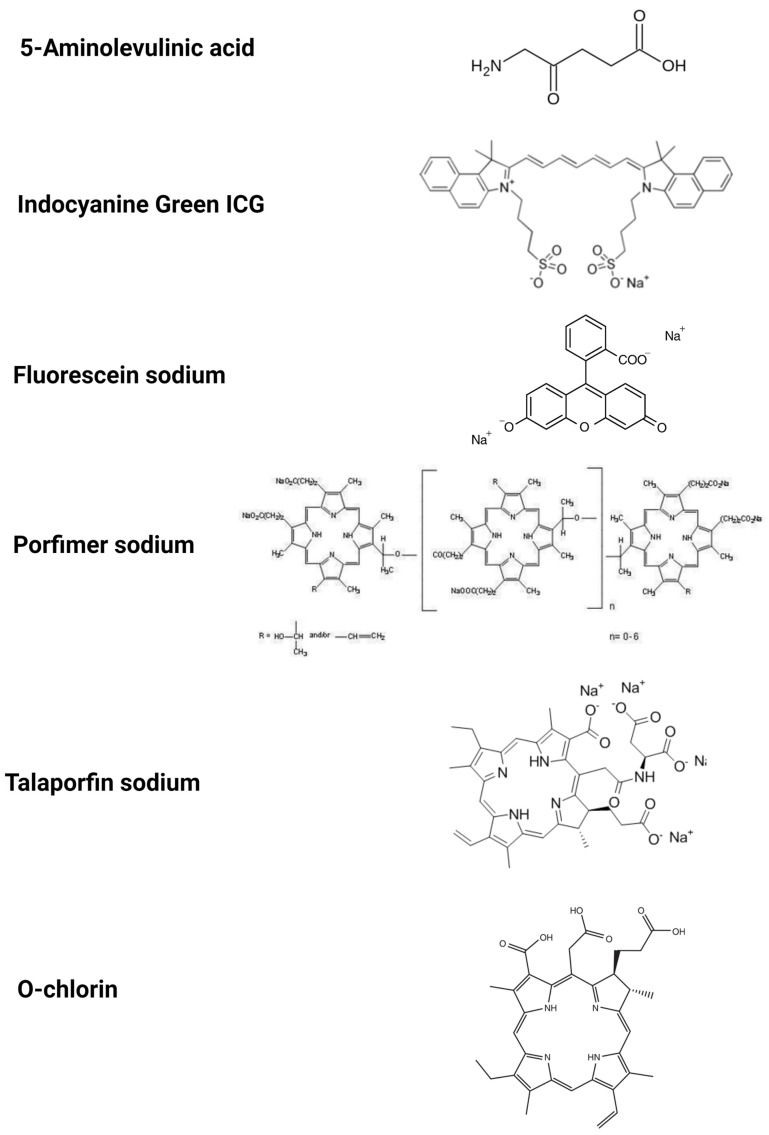
The chemical structure of substances used in photodynamic diagnostics.

5-ALA is a natural amino acid required for heme synthesis; it is a precursor for protoporphyrin (Figure 3). It is a well-known and widely used compound in medicine. It is readily available and registered around the world as a compound suitable for contact with human tissues. 5-ALA and its derivatives are registered and used in PDT of several types of cancer, such as BCC, SCC, and are currently being researched for PDT use in further cancer types. Many medical facilities already have the appropriate equipment for the use of 5-ALA and its adaptation for use in other applications or locations does not involve the need for significant investments. Due to its low toxicity and hydrophilicity and the mechanism in which it is accumulated in cells, 5-ALA can be easily and safely administrated in many ways, including oral, topical, intravenous, intraperitoneal and intravesical routes, depending on where the cancer in need of being visualized is. When administrated, 5-ALA accumulates in cancerous cells, meaning that the synthesis of photoporphyrin-IX increases. After the excitation with blue light (λ = 400–410 nm), the PpIX emits a red–violet light of 635 nm. 5-ALA is already widely used in the detection of several types of tumors, such as glioma, non-muscular invasive bladder cancer [55]. Surgical resection of HGG glioma is preceded by 5-ALA oral administration and during the operation, specialized equipment is used to illuminate cancerous cells for more precise resection [10]. 5-ALA is also used in fluorescent cystoscopy in terms of looking for bladder cancer, although it is being replaced with its more fat-soluble ester, HAL (hexaminolevulinate hydrochloride, Hexvix), that is more effective at accumulating in cells [55]. The dye is administrated into the bladder with a catheter, and after 2–3 h, cystoscopy is performed. This technique is approved and widely used in clinical settings for bladder cancer diagnosis. Because 5-ALA is already widely approved and relatively easy to access, many studies are being conducted to determine its usefulness in other types of cancer. 5-ALA was reported to be useful for the isolation and characterization of circulating tumor-derived extracellular vesicles (EVs) in patients with glioblastoma [54]. Among the limitations are its low specificity (f.e. illumination of inflammation). 5-ALA, despite being widely known and used for the diagnosis of a fixed number of cancerous diseases, continues to be an interesting compound for scientists, and there are currently many studies being conducted to determine whether it is possible to use 5-ALA in the detection of other cancers. Researchers have been testing various cancers in different locations. Several studies have allowed us to eliminate cancers for which 5-ALA-PDD turned out to be ineffective, due to its low specificity. Bronchial malignancies can be used as an example [55]. Tumors that have shown higher specificity and have the potential to be detected with 5-ALA PDD are as follows: oral and larynx cancer [61,62], intracranial meningioma and metastasis, prostate cancer [63], upper and urinary tract tumors [64], hepatocyte cancer, gastric cancer [54,55], endometrium cancer [65], ovarian cancer [66] and peritoneal carcinomatosis [54]. Different types of cancer in the same location may differ in 5-ALA-PDD visualization. As an example, gastric cancers can be mentioned. Intestinal-type gastric cancers and high-grade adenomas show high sensitivity, but diffuse-type carcinomas show much worse sensitivity [54]. Further research is required. Most of the researched cancers have been proven to be able to visualize 5-ALA-PDD; however, common problem occurred:the specificity and sensitivity were lower than desired. A few researchers suggest that further examination of 5-ALA intracellular transportation could lead to the better prediction of cancer visualization [54]. For example, a correlation between PEPT1 and ABCG2 expression and intracellular levels of PpIX has been noted [54] and scientists have suggested that of the determination of PEPT1 and ABCG2 expression can help to predict the effectiveness of 5-ALA-PDD [54].

5-ALA has many drawbacks, which include the following: background autofluorescence and non-specific accumulation in non-cancerous cells; thus, other dyes have been investigated in the hopes of surpassing them.

### 3.2. Indocyanine Green (ICG)

ICG is a water-soluble, anionic, sodium salt and amphiphilic tricarbocyanine molecule (Figure 3). ICG is the next well-known and widely tested compound and it is applied in many medical centres in procedures other than PDD. Additionally, it is cheaper than 5-ALA, which makes it an interesting alternative. A different absorption mechanism and a different excitation and emission wavelength offer the opportunity to investigate new applications. ICG has traditionally been used for vascular perfusion imaging. Common applications include the assessment of perfusion to tissue flaps, retinal angiography, liver clearance test and bowel anastomoses [67]. In these applications, ICG is administrated intravenously right before the procedure and its fluorescence is observed in blood vessels upon reaching the examined area. Its use to visualize cancerous tissues is being researched. To detect tumors, deeper light penetration is required, which was proven achievable with the application of NIR, or narrow-band imaging. The absorption and emission wavelengths are 780 nm and 820 nm, respectively. ICG, when the goal is to visualize cancerous tissues, is usually administrated intravenously sometime before the procedure/operation. When the time is around 24 h before the operation, it is called second-window imaging or TumorGlow [68]. TumorGlow takes advantage of differently built vessels around tumors, which leads to the higher accumulation of ICG in cancerous cells while simultaneously giving normal cells time to excrete ICG, thus lowering false-positive signals [68]. Cancers that have been visualized with the use of TumorGlow are as follows: malignant pleural mesothelioma [69], lung cancer and pulmonary nodules [70], sarcoma [71], anterior mediastinal cancerous lesions [68] and gliomas [72]. The results were good and further research in order to further develop the visualization of these cancers in clinical settings is required. To improve the adherence of ICG to tumor cells, its derivatives were developed (OTL38 and EC17), which are folate analogues combined with NIR dye S0456 and fluorescein isothiocyanate (FITC), respectively [73,74]. Although similar, OTL38 has decreased auto-fluorescence and increased depth of penetration compared to EC17 [73,74]. OTL38 has been tested in the visualization of circulating tumor cells [73], renal cell carcinoma [75], pulmonary metastases [68], gastric adenocarcinoma [76], pituitary adenomas [52] and pulmonary adenocarcinomas [77] and in all the before-mentioned cancers, it shows great sensitivity and specificity. It is also registered for ovarian cancer detection under the name Cytalux [76]. EC17 has been researched in the intraoperative imaging of pulmonary adenocarcinomas and researchers found that EC17 can be used to visualize cancer lesions effectively [77].

TumorGlow, as well as OTL38 and EC17, are currently being clinically tested [70]. The results so far are promising. Scientists suggest further research to widen the use of these dyes and allow them to be clinically used.

### 3.3. Fluorescein Sodium

Fluorescein sodium is one of the compounds that are well-known and have been thoroughly tested and new applications are currently being researched. A completely different accumulation mechanism and cancer labelling method mean that the potentially detected range of tumors with this compound do not align with other substances, the most important of which is 5-ALA. The price of fluorescein sodium is much lower than 5-ALA and the calibration of already existing equipment is easy and cheap. This makes fluorescein sodium a promising compound for PDD.

Fluorescein sodium is a green, hydrophilic crystalline hydrocarbon (Figure 3) that has been tested in theranostic procedures of intrasurgically visualizing glioma for easier resection that is similar to 5-ALA and due to its much lower cost, it is used off-label in this kind of procedure. The mechanism in which fluorescein sodium visualizes brain tumors is different from 5-ALA; instead of accumulating in cancerous cells, it accumulates in the areas where the blood–brain barrier is disrupted and this is the case for near-by tumors. It is administrated intravenously in rather small doses 4 h before the procedure. This compound is characterized by a light absorption maximum at 494 nm and the emission of green fluorescence at 540–690 nm. Fluorescein sodium, due to its different mechanism of visualizing cancerous tissue and its much lower price compared to 5-ALA, is viewed as a promising compound for intraoperarional visualizing of many types of brain tumors. Many studies have been conducted, and it has been tested in the visualization of brain metastases, meningiomas, lymphomas, pituitary, pediatric brain stems, and spinal cord tumors [78]. Due to its advantages, it is already used off-label instead of 5-ALA for high-grade glioma resection [78]. Despite its potential, further research is required to allow its clinical use and determine its use in other cancer detection process.

### 3.4. Photofin (Porfimer Sodium)

Porfimer sodium is another well-known compound and registered for PDT. Porfimer sodium is a sodium salt formed of oligomers up to eight units (Figure 3). Its shorter half-life and water solubility shows the potential to simplify PDD procedures.

Photofrin (porfimer sodium) is a first-generation photosensitizer widely used in PDT for lung cancer, bladder cancer, cervical cancer, non-operative esophageal gastroduodenal cancers, etc. [79]. It was the first FDA-registered photosensitizer for cancer treatment [79]. Porfimer sodium requires 405 nm wavelength excitation and emits light at a wavelength of 630 nm [80,81]. A few studies (mostly performed in Japan) have been conducted to test the possibility of using porfimer sodium in PDD. Namakura et al. tested the porfimer sodium performance in PDD compared to laserphyrin and already clinically used 5-ALA [80]. Porfimer sodium was administered intravenously 12 h before the procedure. The results showed that while porfimer sodium presented fluorescence emission, it was the weakest of the three tested photosensitizers [80]. Lin et al. researched a topical application of porfimer sodium onto BCC, SCC and Bowel disease sites and reported that it could help with the determination of lesion margins [81]. In addition, 3 h after the dye application, the lesions were examined with the use of a proper laser and detector, and displayed bright reddish colors, whereas the surrounding healthy tissue exhibited a blue background. Further histological examination proved the precision of the determination of the margins of the lesions [81]. The researchers determined that cancer detection with photofrin-PDD is possible and effective. Further research is required to fully determine the diagnostic potential of this molecule.

### 3.5. Talaporfin Sodium

Talaporfin sodium is thoroughly tested and used for PDT, mostly in Japan. It is a sodium salt that contains a chlorin group (Figure 3). The fact that it has great water solubility and short half-life differentiates it from other photosensitizers and encourages research for its application.

Laserphyrin (talaporfin sodium) is a second-generation photosensitizer used in intraoperative PDT for malignant brain tumors, as well as lung cancer, early head and neck cancer [79]. Due to its absorption of a longer wavelength than photophrin (664 nm and 630 nm, respectively), laserphyrin enables successful PDT for deeper lying cancerous tissues. Its use in PDD was tested in a limited amount of studies (mostly in Japan) in malignant glioma [82], as well as gastric cancer [80] and in both cases, it was compared to the already clinically used 5-ALA [80,82,83]. The results have proven laserphyrin to be useful and able to compete with 5-ALA. Further research is required to look deeper into the possibilities of laserphyrin-PDD.

### 3.6. O-Chlorin

O-chlorin is a new chlorine derivative with different properties. A completely different cancerous cell biding mechanism gives it the potential to be useful in the visualization of different types of tumors than the currently used and most widely tested photosensitizers, even surpassing them.

O-chlorin, or glycoconjugated chlorin that contains glucose-derived oligosaccharides, such as maltotriose (Figure 3), has been developed to improve the performance of its ancestor, G-chlorin. This compound is proven to be hydrophilic (as opposed to the before-mentioned g-chlorin), and thus is easier to distribute. This molecule takes advantage of the fact that cancerous cells are prone to absorb more glucose and other saccharides than normal cells; thus, the binding of photoactive chlorine with sugar leads to its elevated uptake by cancerous cells. Its usefulness in PDD is currently being evaluated both in vitro and in vivo in mice. The research shows that o-chlorin is characterized by high accumulation in cancerous cells (higher than talaporfin sodium), specifically in lysosomes. In vitro and in vivo tests were performed and proved that O-chlorin accumulates in cancerous cells more specifically than both 5-ALA and talaporfin sodium and has higher cytotoxicity in smaller doses than talaporfin sodium, thus proving that O-chlorin is a promising compound for the future of PDD and PDT [84]. However, more research is still needed.

### 3.7. Raman Spectroscopy—A New Diagnostic Tool Employing Light

Raman spectroscopy (RS) is a new alternative to traditional testing and theranostic approaches using PDT. PDD and PDT, in order to be employed, require the photosensitizer to be administered to the patient’s body, thus creating the possibility of side effects. Raman spectroscopy, on the other hand, has the ability to use very stable molecular probes and, as a result, has the potential to be less toxic [85,86].

The principle of Raman spectroscopy is based on the scattering of light. When the beam of monochromatic light reaches the molecule, it scatters. Most of the scattered light has the same wavelength as the original light; however, approximately 1 in 10^6^ photons scatters inelastically. The energy of the inelastically scattered photons can be higher or lower than the incident one (anti-Stokes and Stokes, respectively). This is called Raman’s effect and the difference between the incoming and inelastically scattered light’s energy is called the Raman shift.

Raman spectroscopy alone allows us to differentiate between alive and dead cells using the ability to recognize the vibrational fingerprints of living cells. By employing correlation spectra and chemometric techniques, it can also distinguish between healthy and diseased cells. The biochemical processes and components of the cells can be visualized as well [85,87,88].

This technique has been tested in the field of cell and biomarker imaging, providing efficient effects. It has been used to distinguish adenocarcinoma from high-grade dysplasia in Barrett’s oesophagus and to visualize changes in cells after radiotherapy. Other cancers that can be diagnosed with Raman spectroscopy include the following: skin cancer, breast cancer and cervical cancer [85,88,89].

There are many Raman spectroscopy-based techniques that differ between themselves in some details. Among them are surface-enhanced Raman spectroscopy (SERS), resonance Raman spectroscopy (RRS), coherent anti-Stokes Raman spectroscopy (CARS) and spatially offset Raman spectroscopy (SORS).

Various nanoparticles have the potential to be excellent probes for Raman spectroscopy. A wide variety of nanoparticles is used for imaging, drug transport and biosensing. Their properties make them (and other molecules labelled with them) well-visualized by Raman spectroscopy. Many nanoparticles have been tested as Raman probes, i.e., small Raman-active molecules that can target biological entities such as cells, tissues, biomarkers, etc. [86,86,90].

Raman spectroscopy can be used for cancer diagnosis using photosensitizers. Brzozek-Pluska et al. have tested the hematoporphyrin intake and distribution in cancerous and non-cancerous breast tissues and proved that it is possible to visualize the difference of this photosensitizer’s intake using Raman spectroscopy. The results showed that the cancerous cells presented higher levels of hematoporphyrin [88]. Horgan et al. [89] researched the theranostic approach system for colorectal cancer. The visualization of the cancerous tissue after the administration of 5-ALA was followed by the PDT procedure after 4 *h* and another evaluation of cancer tissue by Raman spectroscopy. This procedure was proven to be useful and effective [89].

Raman spectroscopy is a new emerging tool for cancer diagnostics and treatment evaluation. It is easy, non-toxic and not invasive, showing potential to be widely used in clinical settings.

## 4. Conclusions

PDD is an innovative and highly valuable technique for cancer diagnostics and treatment. Although currently approved and clinically used photosensitizers such 5-ALA, indocyanine green, sodium fluorescein, porfimer sodium and talaporfin sodium have their limitations, ongoing studies of new and improved photosensitizers are being evaluated and the published results seem promising. Moreover, the integration of nano-PSs, including quantum dots, liposomes, and micelles, in combination with additive or synergistic therapies, are relevant in both preclinical research and future clinical practice. QDs can be successful photosensitizers or energy donors to other photosensitizers that generate ROS after irradiation, leading to cell death during cancer treatment. As many studies show, nano drug carriers for photoactive substances are much more effective in therapies than non-encapsulated substances.

Based on the review of the latest literature, we suggest that both PDD and PDT have the potential to become more frequently used medical methods for diagnostics and cancer treatment. To date, as evidenced by substantial data, the combination of PDT with radiotherapy, chemotherapy, surgery, and immunotherapy is promising.

In conclusion, this review shed light on the latest achievements in PDD and PDT improvements seem to represent the future of the field of oncology.

## Figures and Tables

**Table 1:** Photosensitizers used for PDD in different types of cancer in in vitro and in vivo studies and in clinical trials.

Substance	The Stage of the Research	Types of Cancer
5-Aminolevulinic acid	Registered or clinical use	High-grade glioma Bladder cancer (HAL)
	Clinical trial	EV-tumor-derived extracellular vesicles Brain cancer other than HGG and metastasis to brain Upper urinary tract and prostate Liver cancers Gastric cancers Peritoneal metastasis Gastric cancer
Indocyanine green (ICG)	Clinical trials	Hepatocellular carcinoma (HCC) Colorectal and pancreatic cancer Liver metastases Sentinel lymph nodes in breast cancer and melanoma
EC17	Clinical trials	Glioma Ovarian cancer NSCLC Breast cancer Fr-alfa-rich cancerous cells
OTL38	Registered for clinical use	Ovarian cancer (CYTALUX)
	In vivo—animals	FR-positive metastatic cells etc.—circulating tumor cells Lung cancer Renal cancer Pituitary tumors Gastric cancer
Fluorescein sodium	Off-label clinically used	Glioma
	clinical trial	Brain metastases Meningiomas Lymphomas Pituitary tumors Pediatric brain stem Spinal cord tumors
Porfimer sodium (Photofrin)	Clinical trial	Esophageal cancer Gastric cancer Skin cancer
Talaporfin sodium (contains Ce6)	Clinical trial	Malignant glioma Gastric cancer
O-chlorin	In vivo, in vitro	Yet to be determined

## Data Availability

Not applicable.
